# The genomic scale of fluctuating selection in a natural plant population

**DOI:** 10.1002/evl3.308

**Published:** 2022-12-11

**Authors:** John K. Kelly

**Affiliations:** ^1^ Department of Ecology and Evolutionary Biology University of Kansas Lawrence Kansas USA

**Keywords:** Adaptation, balancing selection, ecological genetics, evolutionary genomics

## Abstract

This study characterizes evolution at ≈1.86 million Single Nucleotide Polymorphisms (SNPs) within a natural population of yellow monkeyflower (*Mimulus guttatus*). Most SNPs exhibit minimal change over a span of 23 generations (less than 1% per year), consistent with neutral evolution in a large population. However, several thousand SNPs display strong fluctuations in frequency. Multiple lines of evidence indicate that these ‘Fluctuating SNPs’ are driven by temporally varying selection. Unlinked loci exhibit synchronous changes with the same allele increasing consistently in certain time intervals but declining in others. This synchrony is sufficiently pronounced that we can roughly classify intervals into two categories, “green” and “yellow,” corresponding to conflicting selection regimes. Alleles increasing in green intervals are associated with early life investment in vegetative tissue and delayed flowering. The alternative alleles that increase in yellow intervals are associated with rapid progression to flowering. Selection on the Fluctuating SNPs produces a strong ripple effect on variation across the genome. Accounting for estimation error, we estimate the distribution of allele frequency change per generation in this population. While change is minimal for most SNPs, diffuse hitchhiking effects generated by selected loci may be driving neutral SNPs to a much greater extent than classic genetic drift.

**Impact Summary**

Across the millions of polymorphisms that reside within most species, how much change is typical from one generation to the next? This is an elementary but important question for a science named after change (evolutionary biology) and has implications across biology from species conservation to medicine (Messer et al., 2016, Rudman et al., 2022, Stearns, 2012). We know the processes that cause allele frequency change within populations (genetic drift, natural selection, migration), but remain largely ignorant about the quantitative pace of genome‐wide evolution. Here, we use population genome sequencing to directly estimate the pace of change, generation‐to‐generation, at millions of loci in a natural plant population. Natural selection is strong but frequently changes in direction. Fluctuating natural selection not only drives loci affecting fitness, but also has a subtle but pervasive effect on the entire genome.

The genetic measure of evolution is Δ*p*, the per‐generation change in allele frequency. In small populations, Δ*p* will be substantial across the genome owing simply to genetic drift. Many species, even those with broad geographic distributions, exist as metapopulations containing many small subpopulations (Wade, 2016, Husband and Barrett, 1992). In large populations however, the importance of drift is greatly diminished and Δ*p* should be minimal on ecological timescales (ca. 10 generations). In contrast, natural selection can generate large magnitudes for Δ*p* in large populations. Strong selection generating significant Δ*p* within populations has been documented for major loci such as color polymorphisms and inversions (Endler, 1986, Ford, 1971, Lewontin and Dunn, 1960, Mérot et al., 2020, Joron et al., 2011), but these examples are often considered atypical given that most genetic variation is generated by loci with smaller phenotypic effects. However, accumulating evidence suggests that selection on hundreds of loci is measurable generation‐to‐generation within natural populations (Messer et al., 2016). Statistically significant ∆*p* at SNPs across the genome has been demonstrated in insects (Bergland et al., 2014, Machado et al., 2021, Soria‐Carrasco et al., 2014), vertebrates (Therkildsen et al., 2013, Chen et al., 2019), and plants (Troth et al., 2018, Anderson et al., 2014, Exposito‐Alonso et al., 2019), both within and between generations.

Genomic scans for selection routinely employ outlier approaches (Lewontin and Krakauer, 1975), finding loci that exhibit features significantly deviant from the genome‐wide distribution. In surveys of nucleotide sequence variation, the features are typically measures of polymorphism, divergence, haplotype structure, or inter‐population differentiation (Luqman et al., 2021, Haasl and Payseur, 2016). In studies of contemporary evolution, selected loci are inferred because they exhibit Δ*p* too large to be generated by genetic drift (Fisher and Ford, 1947, Walsh and Lynch, 2018). Assuming that most polymorphisms are neutral, the genome‐wide distribution for Δ*p* can be used to estimate the effective population size (N_e_) establishing a null distribution for selection tests. This approach is based on the “standard model” of population genetics (Messer et al., 2016) which posits not only that most variants are neutral, but also that their fate is governed by genetic drift. However, selection on a minority of loci can have pronounced effects on neutral polymorphisms owing to processes variously described as hitchhiking (Smith and Haigh, 1974) or linked selection (Cutter and Payseur, 2013) or genetic draft (Gillespie, 2000). The effects of rapid adaptive fixations (selective sweeps (Begun and Aquadro, 1992) and recurrent deleterious mutation (background selection (Charlesworth et al., 1995) on closely linked neutral loci are well appreciated, but the quantitative importance of linked selection (in all its forms) in determining genomewide patterns of variation remains unclear.

In this paper, we characterize genome‐wide Δ*p* over 23 generations (years) within a single large population of the wildflower *Mimulus guttatus* (yellow monkeyflower). We estimated allele frequencies by pooled sequencing of tens of thousands of seeds collected from adult plants at each of the 11 time points, starting in 1998 and concluding in 2021 (Figure 1). While the absolute number of genomes is very large, effective sample sizes are smaller because seeds were collected as maternal families, an average of about 600 distinct families per year (**Methods A**). To accurately estimate allele frequency, we developed a procedure using two independent samples from each year. We first apply Fisher's (Fisher and Ford, 1947) Arcsine Square root transformation ($z\;=\;2\;\mathrm{Si}n^{-1}\left(\sqrt{p}\right)$) and then examine the distribution of differences in z (across all SNPs) between paired samples. The genome‐wide distribution for differences is remarkably normal and homogeneous (Supplemental Figure 3), enabling robust estimation of the error variance from the data itself. Given this characterization of uncertainty, we apply likelihood‐based evolutionary models to predict change through time (**Methods A‐C**). At each SNP, we determined the likelihood of the data under three competing models: Neutral evolution where true change is minimal; Directional selection where frequency changes in a consistent way through time (adapted from Schaffer et al., 1977); and Fluctuating selection with temporally inconsistent change (adapted from Fisher and Ford, 1947). SNP trajectories supporting each of these models are depicted in the lower panel of Figure 1. The first major result of this study concerns the outliers identified from these tests: Strong fluctuating selection is indicated at about 1000 loci distributed across the entire genome. The second major result concerns the background: The overall pattern of allele frequency change through time indicates a pervasive effect of linked selection on neutral polymorphisms.

**Figure 1:**
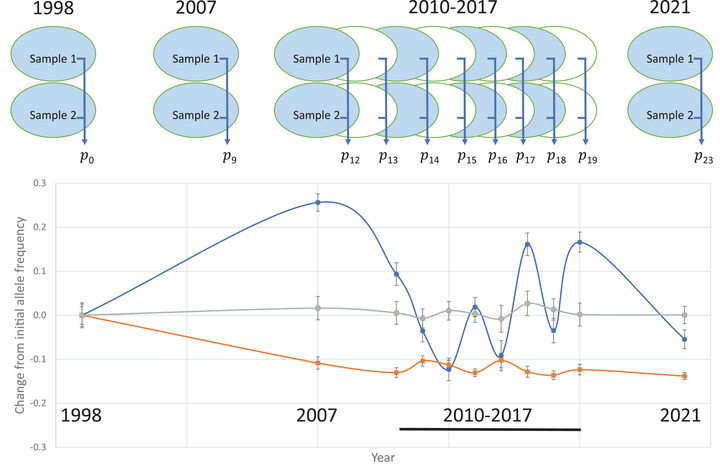
Top: the sampling design to estimate ***p_t_***, allele frequency, from generation 0 (year 1998) to generation 23 (year 2021). Bottom: Examples of SNPs that test positive for Fluctuating (Blue = Chr_ 10, 16339654) and Directional (Orange = Chr_11, 6694754) change versus a Neutral SNP (Grey = Chr_01, 1015040). The error bars are +/‐one standard error.

## Results and Discussion

The study population is located on Iron Mountain (IM) in the Cascade mountains of Oregon (Latitude: 44.402217, Longitude: −122.153317). IM is an outcrossing, annual population with a life cycle restricted to a few months between snow melt and summer drought (Troth et al., 2018, Willis, 1993, Fishman and Kelly, 2015). Seeds were collected each year after all plants had ceased flowering. After DNA extraction and sequencing of two pools per year, we applied stringent filters to putative variants (**Methods A**) and then estimated the allele frequency trajectory for 1,857,010 SNPs. We estimated the census size of IM (the number of plants that flower) at N ≈ 300,000 in the 2013 field season (SI Appendix 1). This number oscillates but the number of flowering plants greatly exceeds 100,000 in most years. Assuming that most SNPs are neutral, we can estimate the effective population size (N_e_) from the overall divergence in allele frequency from 1998 to 2021. Accounting for estimation error (**Methods B**), our procedure yields N_e_ = 11,790. This is much smaller than the census size (discussed below), but we apply this N_e_ to establish conservative significance thresholds for our directional and fluctuating selection tests.

Minimal change is expected for neutral polymorphisms in a large population over 23 generations and most SNPs are consistent with this expectation. Imposing a False Discovery Rate of 5%, we find that 1796 SNPs are significant for fluctuating selection (Supplemental Table 1A), while only 40 SNPs are significant for directional selection (Supplemental Table 1B). Visual inspection of the trajectories confirms that reversals in the direction of change are the rule at SNPs significant for the Fluctuating test. Some of the significant SNPs are closely linked. We calculated Linkage Disequilibrium (LD) among the SNPs in the time series using 165 fully genome‐sequenced lines derived from the IM population (Troth et al., 2018). LD measured as r^2^ (Hill and Robertson, 1968) averages 0.3 to 0.4 within genes, but declines to near zero at inter‐SNP distances of 50kb (Supplemental Table 2), consistent with previous estimates from IM (Puzey et al., 2017). To avoid double counting in the analyses described below, we thin each list to the most significant SNP per gene. This yields 994 “Fluctuating SNPs” and 35 “Directional SNPs”. The Fluctuating SNPs are distributed evenly across chromosomes—there is strong positive linear relationship between the number of genes per chromosome and the number of genes that have a Fluctuating SNP (Supplemental Figure 1). After thinning, the average distance between Fluctuating SNPs adjacent on chromosomes is 254kb.

**Figure 2:**
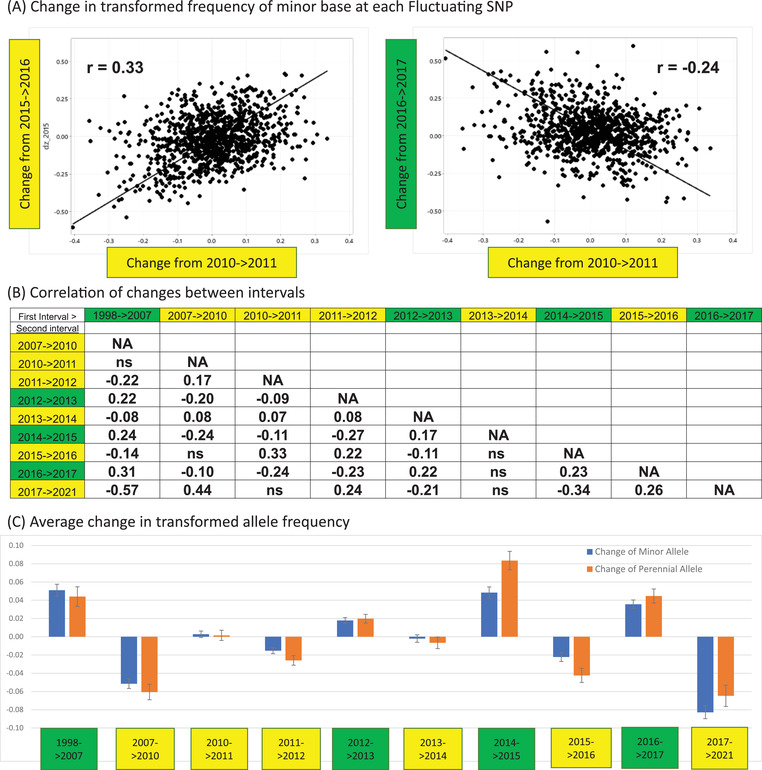
All calculations were performed on transformed frequencies: $z=2\;{\mathrm{Sin}}^{-1}\left(\sqrt{p}\right)$. (A) The contrast of change between two yellow intervals (left) and between a yellow and a green interval (right) for change in minor allele frequency. The lines are orthogonal regressions using the ratio of observed variances for the ratio of error variances. (B) Each significant Pearson correlation (r) of changes between non‐adjacent intervals is reported for the 994 Fluctuating SNPs (p < 0.05; ns = not significant). (C) The average change in transformed frequency of minor (blue, n = 994) and perennial (orange, n = 393) alleles is reported for each interval. Average changes are statistically different from zero except those in 2010‐2011 and 2013‐2014.

**Figure 3:**
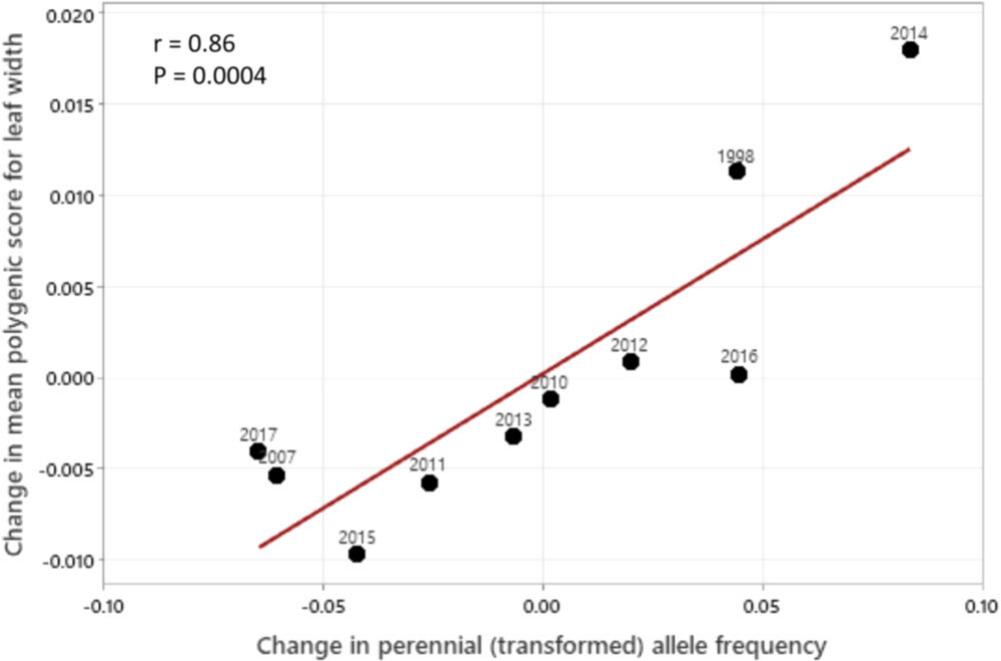
The change in mean polygenic score of leaf width across each of the 10 intervals is predicted from the mean change in Δz(perennial) for that interval (Fig 2C). The observed covariance of changes (3.55E‐04) was greater than 99.96% of permuted cases. The point labels refer to the start year of each interval.

**Figure 4:**
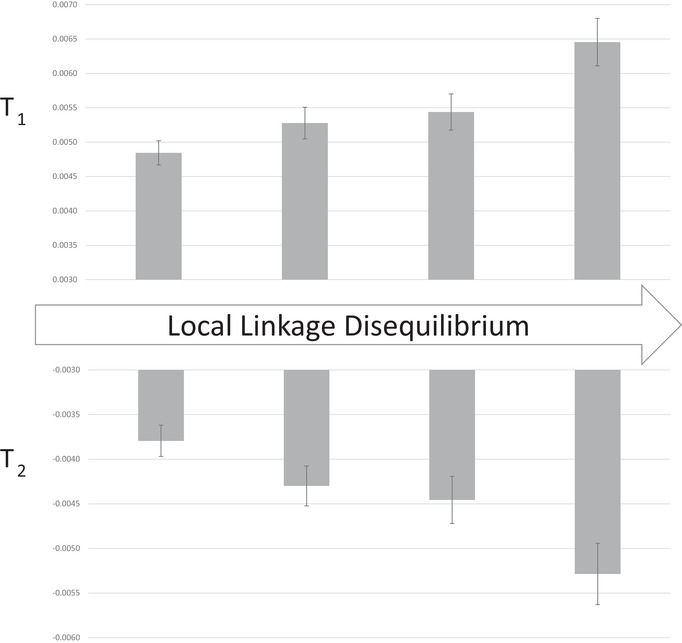
The average of T_1_ and T_2_ in each quartile group of SNPs. SNPs within each group are in the same quartile for standardized linkage disequilibrium calculated on a gene specific basis. The error bars are +/‐ one standard error (the standard deviation of the bootstrap distribution).

**Figure 5:**
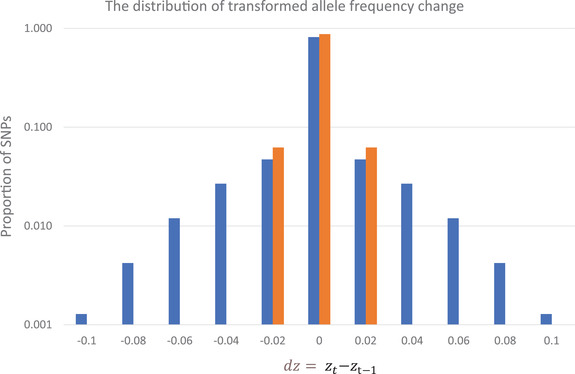
The per generation distribution of *dz* is reported: blue = the ASHR model fit to the real data (2010‐2017), orange = genetic drift with N_e_= 11,790. The bin intervals of +/− 0.01 (0 includes values between −0.01 and 0.01, 0.02 contains values from 0.01 to 0.03, etc). Orange bars for Abs(dz) > 0.03 are less than 10^‐5^ (below the y‐axis minimum). The y‐axis is on a log scale.

If the IM population experienced severe bottlenecks within the 23‐year timeseries, then drift alone could have generated greater fluctuations in some intervals than others. However, this is not a viable explanation for the significance of Fluctuating SNPs. A bottleneck will elevate the magnitude of Δ*p* across the genome. A year‐to‐year analysis of change at the whole genome scale provides no support for such outlier years (Appendix S2). The fluctuating SNPs that emerge as significant in the likelihood ratio tests exhibit much greater change than the genomic background over all intervals. More importantly, drift‐driven changes at neutral SNPs should be uncorrelated between unlinked loci and completely unrelated to the biological attributes of alternative alleles. As shown below, the overall pattern of change at Fluctuating SNPs is remarkably non‐random in both regards (Figures 2, 3, 4, 5).

First, many of the Fluctuating SNPs appear to be responding ***in parallel*** (synchronously) to selection. At any SNP, we can calculate change within each of the 10 intervals between timepoints (1998‐2007, 2007–2010, 2010–2011, etc.). At Fluctuating SNPs, the allele that increased from 2010 to 2011 usually also increased from 2015 to 2016 (*r* = 0.33 in Figure 2A, left). However, this same allele tended to decline from 2016 to 2017 (*r* = −0.24 in Figure 2A, right). The time series for different SNPs are sufficiently synchronized that we can classify intervals into two categories (“green” or “yellow”) that predict change across loci. Figure 2B reports all significant correlations between changes of Fluctuating SNPs between each pair of non‐adjacent intervals. Intra‐category comparisons (yellow versus yellow or green versus green) exhibit positive correlations, while inter‐category contrasts are negative (Figure 2B). In fact, this pattern of correlations defines intervals as green or yellow. The color labels are based on features of the alleles that tend to increase in each sort of interval. The data presented below suggests that alleles that increase in green intervals are associated with early life investment in vegetative tissue and delayed flowering. Yellow alleles are associated with more rapid progression to flowering (more yellow flower tissue relative to green leaves). The exemplar SNP for fluctuating selection (the blue trajectory of Figure 1) matches the green/yellow classification perfectly: The scored allele increased in each green interval and declined in each yellow interval. This is not true of all fluctuating SNPs and not all yellow‐yellow interval contrasts are significantly non‐zero (Figure 2B). For interval correlations, we excluded adjacent intervals because the shared parameter estimate induces a statistical correlation between estimates. Non‐adjacent intervals are based on entirely distinct data and should exhibit no correlation under drift.

Second, the pattern of change at Fluctuating SNPs is predictable from the properties of alternative alleles, specifically their frequency within the IM population, their distribution among populations of *M. guttatus*, and their association with phenotype. Minor alleles (the less common variant) tend to increase in green intervals but decline in yellow intervals (blue bars in Figure 2C). Only two intervals, 2010–2011 and 2013–2014, fail to exhibit a significant change in minor allele frequency. For characterizing patterns across intervals, it is useful to calculate a simple index of change for each SNP: $C_{g}=\left(\mathrm{Sum}\;\mathrm{of}\;\mathrm{changes}\;\mathrm{in}\;\mathrm{green}\;\mathrm{intervals}\right)\;-\left(\mathrm{Sum}\;\mathrm{of}\;\mathrm{changes}\;\mathrm{in}\;\mathrm{yellow}\;\mathrm{intervals}\right)$.

$C_{g}\;$will be positive if the reference base tends to increase in green intervals but decline in yellow, negative if the alternative base follows this pattern. The distribution of $C_{g}$ among Fluctuating SNPs is clearly bimodal (Supplemental Figure 2), but SNPs that do not follow the green/yellow pattern yield $C_{g}\;$ close to zero. We find a strong, negative relationship between $C_{g}$ and the average frequency of the reference base within IM ($r\;=\;-0.38,\;p\approx 10^{-38}$). When the reference base is less common than the alternative base, it is usually green. It is typically yellow when the major allele. This does not imply that selection is inherently frequency dependent, just that there is a strong association between current population frequency and the pattern of change since 1998.

The SNPs present IM are polymorphic within other populations across the species range (Monnahan and Kelly, 2017). *M. guttatus* occurs in both annual and perennial forms. Populations exhibit adaptive genetic differentiation associated with this difference (Hall and Willis, 2006), but there is clear evidence of ongoing gene flow between annual and perennial populations (Lowry and Willis, 2010, Colicchio et al., 2020). To identify loci distinguishing annual and perennial ecotypes, Gould et al. (2017) collected plants from 47 perennial and 50 annual populations and then sequenced a pooled DNA sample for each group. We here remapped the sequence data from the annual and perennial pools to the current *M. guttatus* reference genome and then estimated allele frequency within the pools for the time series SNPs (see **Methods D**). Among the 994 Fluctuating SNPs, 963 have at least 20 sequence reads in both Annual and Perennial samples and thus can be used to obtain a rough estimate of allele frequency. Among these, 393 SNPs exhibit sufficient divergence between pools to label one allele as perennial and the alternative as annual. Considering this classification in relation to the time series, we find that perennial alleles tend to increase in green intervals and decline in yellow intervals (orange bars in Figure 2C). Next, we calculated the simple difference in transformed allele frequency (perennial pool minus annual pool for all 963 SNPs). We find a highly significant positive association between $C_{g}$ and this difference ($r\;=\;0.14,\;p\approx 10^{-5}$), confirming the pattern evident from the 393 SNPs with high divergence (Figure 2C). The responses of perennial alleles and minor alleles are distinct but not independent. The perennial allele is the minor allele in 248 of 393 cases (63%).

Next, we tested whether the phenotypic effects of alleles predict their fluctuations through time. The association of allelic features with direction of change (Fig 2) indicates an effect of natural selection, but not how fitness differences are generated. We can estimate the association of Fluctuating SNPs with phenotypes using data from the genome‐wide association study of Troth et al. (2018). We first remapped the sequence data from the 165 homozygous lines with greenhouse phenotype data to the current genome assembly. The sequenced lines are all derived from IM and we find that allele frequency within the lines is very strongly correlated with the mean allele frequency from the time series (r = 0.97 at Fluctuating SNPs), which indicates the lines are a representative sample of the IM population. We next re‐estimated SNP effects on traits (two phenology statistics and 11 measures of plant/flower size) using a linear mixed model (**Methods E**). This produced an estimate (and standard error) for the additive effect of Fluctuating alleles on each trait. We could then calculate a year‐specific mean “polygenic score” for each trait: ${\bar{P}}_{t}=\sum_{i}2p_{i,t}\alpha_{i}w_{i}\;$, where i indexes SNPs, $p_{i,t}$ is the frequency of the reference allele at SNP i in generation t of the timeseries, $\alpha_{i}$ is the additive effect of this allele, and $w_{i}$ is an SNP‐specific weight determined by the standard error on $\alpha_{i}$. If additive effects were estimated without error, ${\bar{P}}_{t}$ would be the contribution of Fluctuating SNPs to the mean breeding value (and thus mean phenotypic value) of the trait (Walsh and Lynch, 2018). However, all the traits are highly polygenic (Troth et al., 2018) and estimation error on per‐locus effects is significant. Thus, ${\bar{P}}_{t}$ averages across SNPs using generalized least squares giving greater weight to SNPs with more accurate estimates (**Methods E**). These weights do not change with time so differences in ${\bar{P}}_{t}$ though time are driven entirely by changes in allele frequency.

We find a strong and highly significant association between the change in mean score ($\Delta \bar{P}$) of Leaf width and whether an interval is green or yellow (Figure 3). We used the change in Perennial frequency (Fig. 2C) as a continuous measure of green/yellow, but equivalent results are obtained if minor allele change is used instead (Supplemental Table 3). Across Fluctuating SNPs, alleles that increase leaf size increased (on average) in green intervals and declined in yellow. The same pattern is observed for phenology and flower size measures, although permutation tests proved only marginally significant (0.01<p<0.05) or marginally non‐significant (0.05<p<0.10) for these traits (Supplemental Table 3). Critical here, leaf width and flower size were measured *on the day plants first flowered*. Previous genetic studies of the IM population demonstrated a genetic trade‐off between development rate and size at reproduction (Troth et al., 2018, Kelly, 2008). Genotypes that delay flowering usually have greater above‐ground biomass (both leaves and flowers) when they do flowering. Genotypes that progress to flower more rapidly are typically smaller plants at this stage. At the loci mapped in their study, Troth et al. (2018) showed that “small/fast” alleles are usually more frequent than the alternative “large/slow” alleles within IM. Consistent with this, we here find that minor alleles tend to increase in green intervals and decline in yellow intervals (Figure 2C) and that green alleles are associated with within increased vegetative mass when plants reach flowering (Fig 3).

### THE MAINTENANCE OF POLYMORPHISM WITH FLUCTUATING SELECTION

SNP variation within IM is sampled from the *M. guttatus* metapopulation (the same polymorphisms segregate in other populations (Monnahan and Kelly, 2017)); a fact immediately relevant to the maintenance of polymorphism. We identified 20x more Fluctuating SNPs than Directional SNPs, which is unsurprising given that strong directional selection eliminates polymorphism rapidly – they cease to exist when the favored allele fixes. Single locus population genetic models predict fairly stringent conditions for balanced polymorphism (Hedrick, 1976), but multi‐locus models indicate that fluctuating selection can greatly elevate the genetic variance if novel variants are occasionally introduced into the population (Bürger and Gimelfarb, 2002, Kondrashov and Yampolsky, 1996, Wittmann et al., 2017). These models posit mutation as the input of new variation, but gene flow is the principal source of novel alleles into IM (Puzey et al., 2017). While immigration is not high in an absolute sense (IM exhibits Fst ≈ 0.1 with neighboring populations (Monnahan et al., 2015)), it delivers variation at a rate that is orders of magnitude higher than mutation.

Considering quantitative variation, the size/speed tradeoff evident within IM is also evident among populations. Most populations, particularly those at lower latitudes and elevations, have longer growing seasons than IM. This allows greater investment in vegetative growth prior to flowering. In areas where the ground remains wet year‐round, *M. guttatus* is often perennial. Reciprocal transplant experiments between annual and perennial populations of *M. guttatus* have demonstrated divergent selection with early flowering favored in the annual habitat and delayed flowering (combined with greater vegetative mass at the time of flowering) in perennial habitats (Hall and Willis, 2006). In this way, the intra‐population green/yellow distinction identified by the time series data matches the annual/perennial divergence pattern – perennial alleles tend to increase in green intervals (Figure 2C) and green alleles exhibit the phenotypic tendencies of perennial populations.

A chromosomal inversion on chromosome 8 segregates within *M. guttatus* and the derived orientation tends to predominate in perennial populations (Lowry and Willis, 2010, Twyford and Friedman, 2015). This inversion occurs only at very low frequency at IM, if at all (Monnahan and Kelly, 2017). However, many SNPs within this section of the genome segregate within IM and exhibit allele frequency difference between annual and perennial orientations (Monnahan and Kelly, 2017). About 40 of the Fluctuating SNPs occur in the inverted section of chromosome 8. The Kirkpatrick and Barton (2006) model predicts that selection can favor inversions if they impede recombination between linked loci subject to geographically varying selection; in this case between habitats allowing prolonged growth versus those that require rapid progression to flowering. Of course, the great majority of Fluctuating SNPs are not in the inversion region of chromosome 8. For this reason, the Annual/Perennial classification used for Figure 2C should be considered a marker of life history variation among populations. Small/fast alleles *at loci across the genome* are likely to be at higher frequency in annual populations than perennial populations given the highly polygenic basis of variation (Troth et al., 2018).

### THE GENOMEWIDE EFFECT OF FLUCTUATING SELECTION AND THE PACE OF CHANGE

The fluctuating SNPs provide a clear indication of selection on individual loci, but we expect that selection on many loci to have diffuse effects on polymorphisms throughout the genome via linked selection (Gillespie, 2000, Barton, 2000). Buffalo and Coop (2020) developed a method to evaluate linked selection in time series data by partitioning the variance in total change into components. Considering transformed allele frequency (z), the relevant equation is:

$$\begin{array}{cc} \mathrm{Var}\left[z_{t}-z_{0}\right]= & \sum_{i}\mathrm{Var}\left[\Delta z_{i}\right]+\sum_{i}\sum_{j\neq i}\mathrm{Cov}\left[\Delta z_{i},\Delta z_{j}\right]\\ = & T_{1}+T_{2} \end{array}$$

where$\;\Delta z_{i}$ is change within a particular interval (say 2007 to 2010) and the summations are taken over all intervals. T_1_ is the sum of variance for changes within intervals. T_2_ is the sum of covariances for all pairwise comparison between distinct intervals.

Perhaps the most fundamental feature of evolution by genetic drift is that the direction and magnitude of Δ*p* in the current generation is unaffected by change in past generations. Change in the present has no effect on future generations. For these reasons, T_2_ should be zero under drift. In contrast, hitch‐hiking of neutral alleles linked to selected loci can generate correlations across generations (Robertson, 1961). $Cov[\Delta z_{i},\Delta z_{j}]$ will be positive if the same selected allele increases in each interval. With fluctuating selection however, we expect that $Cov[\Delta z_{i},\Delta z_{j}]$ will be variable and often negative. After accounting for estimation error (**Methods F**), we find that $Var\;[z_{t}-z_{0}]=\;0.00104$ across all SNPs in our time series. Partitioning this variance, T_1_ = 0.00551 and T_2_ = −0.00446. T_2_ is significantly negative with a 95% confidence interval (−0.0048, −0.0042) clearly bounded away from zero.

The significantly negative T_2_ implies that the average SNP, which presumably has little or no effect on fitness, exhibits a slight but highly significant inter‐generation covariance. Change in the current generation tends to cancel changes in previous generations. Consequently, there is less total change in frequency over the full span of 23 generations than expected from the magnitude of single‐generation changes (characterized here by T_1_). Considering the specific contrasts between intervals (the components of T_2_), most contrasts are significantly different from zero (correcting for bias introduced by estimation error in allele frequency, see **Methods F**). They fluctuate from positive to negative depending on the contrast (Supplemental Table 4). As a comparison to the calculations made using all SNPs, we calculated $Cov[\Delta z_{i},\Delta z_{j}]$ specifically for the Fluctuating SNPs for all time intervals. As expected, there is a positive relationship between covariances based on the Fluctuating SNPs and the whole genome, but the magnitudes are much larger for Fluctuating SNPs (Supplemental Figure 4).

Hitch‐hiking of neutral variants with selected loci should be most pronounced when there is reduced recombination (Barton, 2000). Recombination rate varies across the Mimulus genome (Flagel et al., 2019) and we here use local LD estimates as a proxy for local recombination rate. For each gene, we calculated a standardized measure for LD which accounts for the variation in inter‐SNP distances within genes (**Methods E**). Next, we calculated T_1_ and T_2_ specific to each gene. Standardized LD exhibits a highly significant positive association with T_1_ (r = 0.050, p<0.001) and a highly significant negative association with T_2_ (r = −0.047, p<0.001). Figure 4 illustrates this association by subdividing genes into four quartiles (lowest linkage disequilibrium to highest) with an equal number of SNPs in each group. The amount of change per generation (T_1_) as well as the extent of canceling between generations (T_2_) are greater in genomic regions that have stronger associations between alleles.

The overall distribution of allele frequency change per generation can be inferred from our time series by separating estimation error from true differences in allele frequency. This is impossible for any particular SNP, but feasible for the entire distribution. We obtained a reliable standard error for each SNP/interval via the paired sample design (Figure 1), and given these, Stephens' (2016) empirical Bayesian approach (ASHR) can be used to extract the underlying distribution for “true effects.” Here, true effect is the change in transformed allele frequency at an SNP over one generation ($dz\;=\;z_{t}-z_{t-1}$; **Methods F**). We exclude multigeneration intervals, for example, 1998–2007, because of the non‐independence of change across generations (Figure 4).

ASHR estimates the distribution for *dz* as a mixture of normal density functions (Supplemental Table 5). For this dataset, most of this mixture is SNPs with no change (33%) or very little change (44%); the latter category having a standard deviation of 0.004 for *dz*. For the full distribution, about 82% of SNPs have an absolute *dz* < 0.01 per generation (Figure 5). For untransformed allele frequency (p), this corresponds to a change of less than 0.005 from an initial p of 0.5. If we consider our estimate that N_e_ = 11,790, pure genetic drift will produce an absolute *dz* < 0.01 about 88% of the time, which is only slightly greater than the 82% from ASHR. The remaining 23% of SNPs in the estimated mixture distribution for *dz* have larger standard deviations (about 0.035 on average). These SNPs generate heavy tails in the distribution (Figure 5). About 9% of the observed distribution has Abs(*dz*)>0.03, an outcome that occurs only once every 250,000 generations within neutrality.

Charlesworth (1996) argued that one of the major objectives remaining for evolutionary genetics is to characterize the distribution of selective effects on mutations. Figure 5 is a related but distinct entity. The distribution of *dz* (allele frequency change) is the realization of selection and drift (and immigration in populations where it occurs at a sufficient rate). While the distribution for selection coefficients is often treated as a set of constants (one value for each locus), *dz* is not constant within loci. The change at locus may have a fixed expectation in some cases, for example in a closed population with no selection (the mean is zero), but the actual change per generation will always vary through time. In this dataset, we find that the variance of estimated *dz* is remarkably uniform over allele frequency (Supplemental Figure 3), which reflects the effectiveness of Fisher's Arcsine Square root transformation in reducing heteroscedasticity. Importantly, this study excluded SNPs where the minor allele was ≤0.05 in frequency. There are a very large number of rare‐allele polymorphisms within IM (Brown and Kelly, 2020) and the distribution of selection coefficients on these loci (as well as the distribution of change) must be quite distinct from the corresponding distribution for intermediate frequency variants. At least for intermediate variants however, our tests indicate that selection cannot be effectively characterized by a single constant coefficient per locus.

## Outstanding Questions

What environmental agents generate fluctuations in selection? Synchronous changes in allele frequency could be driven by biotic or abiotic factors (Rudman et al., 2022, Decaestecker et al., 2007, Lively, 2010). Our timeseries for a SNP is a multi‐normal vector, and therefore, it is straightforward to test environmental variables as predictors of change. In this study, power is limited by the small number of intervals (10 per SNP). This can be addressed through more intensive sampling of a single population or by considering multiple populations simultaneously (Machado et al., 2021). Direct manipulation of environmental variables, followed by fitness measurements on individuals or monitoring of entire populations within experimental evolution studies, may be required to establish causality of selective agents (Rudman et al., 2022, Mitchell‐Olds and Shaw, 1987, Rennison et al., 2019). Resurrection studies provide a powerful means to exploit “natural experiments” to understand the phenotypic response to selection (Kooyers et al., 2021, Franks et al., 2016).

How many loci are under selection in the IM population? We identified about 1000 genes that contain significant Fluctuating SNPs. In most regards, our procedures are conservative. For example, we considered only bi‐allelic SNPs, excluded any SNP that deviated between individual and pooled sequencing, and excluded all inter‐genic SNPs (**Methods A**). Selection on intergenic variants would be detected only if the loci were in strong LD with genic SNPs. Counter to these arguments, there are several ways that selection on a few hundred loci could potentially generate significant tests in 1000 genes. Considering hitchhiking, we found minimal LD among our Fluctuating SNPs, but that was estimated from sequenced lines that are >6 generations of self‐fertilization removed from the sampled field plants (Troth et al., 2018). Recombination in the first few generations of line formation could have eliminated moderate LD among loci that are not closely linked (Gompert et al., 2022). Diffuse LD is generated as a direct result of selection on a quantitative trait and also when immigration introduces divergent haplotypes into a population. A more subtle explanation for excess significance is our null hypothesis that neutral SNPs are evolving by genetic drift. The covariance of changes across generations (Figure 4), an indicator of linked selection, undermines the null likelihood model which assumes independent changes with each generation. To some extent, the effects of linked selection are absorbed into our estimate for N_e_ and the Fluctuating SNPs are outliers relative to the distribution of change generated by this N_e_ estimate. Also, hitchhiking arguments do not explain why the biological attributes of alternative alleles, such as their effects on phenotype (Figure 3), predict patterns of change at fluctuating SNPs.

Beyond Mimulus, we suggest that the distribution of allele frequency change (e.g., Figure 5) should be a target for empirical characterization within natural populations of many species. In this study, the amount of change at most SNPs was not large in absolute terms, but still greater than what one would expect given the large number of plants flowering in the population. The genomewide effect of linked selection may explain why N_e_ estimated from allele frequency change (11,790) is an order of magnitude lower than the observed number of reproductive plants. Perhaps more obviously, about 1000 loci across the genome exbibit evolution that clearly stands out from this background of drift and linked selection. The study of contemporary evolution certainly has limits. The zero bin of Figure 5 contains not only neutral SNPs but also those under weak selection. Weak selection may be indistinguishable from drift on ecological time scales but still yield very distinct evolutionary outcomes over long time periods (Ohta, 1976). However, a scale up of the method employed here (in number of populations, number of individuals sampled per generation, and number of generations scored) could provide an increasingly precise characterization of evolutionary forces in a great diversity of taxa.

## Materials and Methods

### A. SEED COLLECTIONS, SEQUENCING, AND VARIANT IDENTIFICATION

Each field collection was conducted after flowering had ceased at the site and all adult plants were desiccated (in July or August). Among all plants that had produced fruit, a random sample was harvested with all seeds from each plant collected into an envelope. The number of maternal families varied from year‐to‐year and are reported in table.

**Table:** 

Year	Total Maternal families
1998	494
2007	1500
2010	340
2011	777
2012	713
2013	752
2014	215
2015	400
2016	478
2017	571
2021	500

Except for 2021, these collections were conducted as part of other studies (Fishman and Kelly, 2015, Kelly, 2008, Fishman and Willis, 2008, Lee et al., 2016, Nelson et al., 2019). To make sequencing libraries, we randomly sorted maternal families into two groups within each year (Samples 1 and 2 in Figure 1). We took an approximately equal sample of seeds from each envelope (maternal family) within a year to form the seed pools. We sampled ca. 20 seeds per envelope from years with high fecundity, but only 10 per envelope in years with lower mean fecundity. This adjustment ensured a more nearly equal contribution per family within each year. This difference might have inflated the estimation error for allele frequencies in years with 10 instead of 20 seeds per family, but as shown below, other factors proved more important.

Allele frequency within each sample is the average across maternal families. The DNA from each Mimulus seeds is predominantly embryo (Elen Oneal, personnel communication), equally composed of maternal and paternal contributions. The maternal contribution is from a single diploid genome while the paternal may be a mixture of multiple genomes depending on the extent of multiple paternity. Given random sampling of plants, the average across families provides an unbiased estimate of allele frequency. However, the magnitude of estimation error is more difficulty to specify *a priori*. The error variance depends now only how many maternal families are sampled and the number of seeds per family, but also on how many sires are sampled per family and on how evenly seeds contribute DNA to the pool (always a factor with pooled sequencing). Given uncertainty on these latter processes, we apply a non‐parametric procedure (described below) in which the data itself estimates the error variance. This procedure requires only two independent samples of the same population.

We extracted DNA from each seed bulk by pulverizing the seeds on liquid nitrogen in a mortar and used the Nextera DNA Flex kit to make genomic libraries. In addition, we grew 48 individual plants from the 2021 collection and extracted DNA from each.  We made a sequencing library from the DNA of the individual plants using the Swift 2S Turbo DNA Library Kit and sequenced each library with Illumina PE 150 reads. All sequencing was done as part three distinct S4 Novoseq runs at the University of Kansas medical center genomics core. For all samples, we cleaned and trimmed the paired reads using fastp (Chen et al., 2018) with these settings: –cut_mean_quality 30, –cut_window_size 2, and –length_required 50. We then mapped reads to the V5 reference genome using the bwa (v0.7.17) mem command (Li, 2013) and to sorted resulting bam files using samtools version 1.9.  Next, we used picard tools (https://github.com/broadinstitute/picard) to remove duplicates and add read groups, and indexed the output bams using samtools (Li et al., 2009).  On the full collection of samples (the pooled seed libraries, the individuals from 2021, and the pooled samples from Gould et al. (2017)), we called variants using bcftools (version 1.9) mpileup with the output piped to bcftools call (Li, 2011).  SNP calling was run in parallel applied to each 100kb section of the *Mimulus guttatus* reference genome (https://phytozome‐next.jgi.doe.gov/info/MguttatusTOL_v5_0).  These steps were implemented using the programs clean.split.fqs.py, map.split.fqs.py, merging.py, rmdups.index.py, and call.snps.bcf.py in sequence.  These programs were written in Python 2.7 as were those described below.  All programs are contained in Supporting Information S1.

We concatenated the VCF files and retained all bi‐allelic SNPs with a minimum mapping quality (MQ) of 20 and a min QUAL score of 20.  Next, we classified all SNPs as “genic” (within a gene of the v5 annotation) or “intergenic” and determined the count of reads at each SNP in the population pools for each category.  The median depth for genic SNPs (12,246) was much greater than for intergenic (6,095).  This difference is unsurprising given previous sequencing studies of *M. guttatus* showing that read mapping is routinely unreliable outside of genic regions (Troth et al., 2018, Monnahan et al., 2021).  For this reason, we focused all subsequent analyses on genic SNPs.  We used the AD field of the vcf to estimate reads carrying the reference and alternative base, respectively. Given these counts, we extracted the read depth (m) and transformed allele frequency (z) for each SNP with minor allele frequency ≥ 0.05 (based on an average of untransformed allele frequency estimates across all pools), and a total depth (across samples) between 9500 and 16000.  These steps were conducted using the programs clean.vcf.py, vcf.readcounts.py, bulk.depth.py, and m_and_z.genic.py.

Next, we calculated υ, the null variance (Kelly and Hughes, 2019) for each pair of samples (Figure M1). This is based on averaging the observed values for ${\left(z_{1}-z_{2}\right)}^{2}$, noting the predicted read depth variance for each SNP ($\frac{1}{m_{1}}+\frac{1}{m_{2}})$. We first used the set of υ values for each pair of samples to identify outlier SNPs.  We suppressed SNPs that exhibit excessive divergence between paired samples of the same population because each sample is estimating the same true allele frequency.  Under our model, this divergence should be normal with mean zero and variance ($\upsilon +\frac{1}{m_{1}}+\frac{1}{m_{2}}$).  If we square the (difference /standard deviation) within each year, and then sum across years, the resulting quantity should follow a chi‐square distribution with degrees of freedom equal to the number of years (paired samples).  We performed this test on each SNP and excluded any SNP with a p‐value < 0.01.

We also extracted the mean and standard error (SE_1_) of z from the 2021 pools to compare to the individual genome sequences of plants from that year.  We calculated z from the individual samples and a standard error (SE_2_) on this estimate and calculated a t‐statistic based on these numbers: $t\;=\frac{\left(z_{pool}-z_{individual}\right)}{\sqrt{SE_{1}^{2}+SE_{2}^{2}}}\;$. We suppressed any SNP where ABS(t)>3.0.  After imposing all these filters, we recalculated the null variances on the remaining SNPs.  These steps were implemented using the programs Null.var.estimation.py, outlier.snps.py, Reduce.snpset.v2.py, and z.per.year.v2.py, in sequence. The estimated υ from each year is reported in the table below. The effective number of genomes is the sample size with perfectly even contribution of each genome to the pool (= 2/υ).

**Table:** 

Year	υ	Effective number of diploid genomes
1998	0.00857	233
2007	0.00612	327
2010	0.00619	323
2011	0.00294	680
2012	0.00215	931
2013	0.00343	584
2014	0.01071	187
2015	0.00896	223
2016	0.00627	319
2017	0.00469	426
2021	0.00533	375

This table illustrates that sampling fewer seeds per family in low fecundity years (2010, 2012, and 2021) did not unduly inflate estimation error. The highest values for υ are observed in years with lower numbers of maternal families (2014 and 2015).

### METHODS B: EFFECTIVE POPULATION SIZE ESTIMATION

There is a considerable literature on estimation of N_e_ from allele frequency time series (reviewed in Ch 4 of Walsh and Lynch (2018)). Here, we use a robust estimation procedure based on transformed allele frequencies from the first (1998) and last (2021) samples. We applied Fisher's angular transform to allele frequencies, both for N_e_ estimation and tests for selection (**Methods C**) because it greatly simplifies all analyses. For all SNPs, the change from beginning to end $\left({\bar{z}}_{2021}-\;{\bar{z}}_{1998}\right)$ is normally distributed with mean zero and variance:

$$\mathrm{Var}\left[{\bar{z}}_{2021}-{\bar{z}}_{1998}\right]=\frac{t}{2N_{e}}+\mathrm{Var}\left[{\bar{z}}_{1998}\right]+\mathrm{Var}\left[{\bar{z}}_{2021}\right]$$

Here, t = 23 and $Var\left[{\bar{z}}_{2021}\right]\;and\;Var[{\bar{z}}_{1998}]$ are known constants for each SNP (**Methods A**). It is simple to calculate the average estimation error variance, $\sigma_{x}^{2},$ across all SNPs. We can then use the overall distribution of $\left({\bar{z}}_{2021}-\;{\bar{z}}_{1998}\right)$ to infer N_e_. While the distribution is remarkably normal in general (Supplemental Figure 3), the variance could be inflated by outlier loci under selection. For this reason, we estimate $Var[{\bar{z}}_{2021}-\;{\bar{z}}_{1998}]$ from the interquartile range (IQR) of the distribution, which is less sensitive to outliers. It follows that ${\hat{N}}_{e}=\frac{t}{2b}\;$, where $b\;=\;{\left(\frac{IQR}{1.34896}\right)}^{2}$ ‐ $\;\sigma_{x}^{2}$. The resulting calculation yields ${\hat{N}}_{e}=\;11790$ and was performed using the program Ne.robust.estimation.py.

For this analysis, we used data only from the first and last samples to estimate N_e_ , a choice based on the specific features of this dataset. Intermediate samples in a time series can be incorporated into N_e_ estimation (e.g. (Jónás et al., 2016) and references therein), but here the great majority of the signal for N_e_ comes from 1998 to 2021. These samples were sequenced at greater depth than intermediate samples and thus have lower $\sigma_{x}^{2}$. Also, the drift signal for this contrast, $\frac{23}{2N_{e}}$, is much greater than for most consecutive intervals. In fact, for a couple of intervals, the divergence IQR is very slightly less than predicted by estimation error alone, which yield ${\hat{N}}_{e}=\;\infty$. Still, a robust z‐based estimator for full time series is useful objective for future method development.

### METHODS C: MODELS OF ALLELE FREQUENCY CHANGE

We implement the “Fisher–Ford” test (see Walsh and Lynch (2018), pp 272–275) after revising the procedure to take data from two pooled samples per year.  Testing for allele frequency change is straightforward since the vector of ${\bar{z}}_{j}$ values for a SNP (j indexes year) are multi‐normal and the (co)variance matrix for this vector is a set of known constants. The variance for sample i is: 

$$V_{ii}=\frac{t_{i}}{2N_{e}}+Var\left[{\bar{z}}_{i}\right]$$

where $t_{i}$ is the number of generations from time 0 for sample i, $N_{e}$= 11790, and $Var[{\bar{z}}_{i}]$ was determined for each SNP in Methods A (Figure M1). The covariance between sample i and a later sample j is:

$$V_{ij}=V_{ji}=\frac{t_{i}}{2N_{e}}$$

The likelihood of the data under drift versus selection depends on how the E[${\bar{z}}_{j}$] are specified. If evolution occurs by genetic drift E[${\bar{z}}_{j}]\;=\;\mu_{0}$ for all j (a single parameter to be estimated from the data). μ_0_ is the true population value for z at t = 0. Changes in ${\bar{z}}_{j}$ through time are accommodated by the variance/covariance matrix.  Under the most general alternative model, which is our Fluctuating selection model, each sample is characterized by its own parameter: E[${\bar{z}}_{j}]\;=\mu_{j}\;$.  Since we have 11 time points, the Fluctuating selection model has 10 more parameters than the drift model and so the likelihood ratio test has 10 degrees of freedom (df).

The Directional test, proposed by Schaffer et al. (1977), is a special case of the Fisher‐Ford test. Selection is admitted by estimating a constant for beginning (μ_0_) and end (μ_23_) of the time series and positing that the mean changes linearly with time between these two points.  This model has two parameters and thus the likelihood ratio test (directional versus drift) has 1 df.  The MLE for parameters have analytical solutions for all three models (Walsh and Lynch, 2018) so calculations are very fast. Both tests were applied to all SNPs with data in all 11 years of the time series using the program linear.and.fluctuating.tests.py. As a check on the validity of our p‐values from these tests, we performed forward simulations with drift acting independently on all ≈1.86 million SNPs. The sample sizes and read depths per SNP matched the actual study. We performed 1000 replicate simulations using the program simulate.drift.two.tests.py, and then extracted the p‐values at each simulated SNP within each replicate. Next, we determined the mean number of tests per replicate to pass successively smaller p‐value thresholds and aligned these numbers to the actual p‐value distribution from the real data. This establishes an empirical False Discovery Rate (Supplemental Table 6). The FDR cutoffs obtained by this method are nearly identical to those from the standard Benjamini‐Hochberg Procedure (Benjamini and Hochberg, 1995). The latter was obtained by applying the p.adjust() function in R directly to the p‐values obtained from tests on real data. Given p‐value thresholds for 5% FDR for each test tests, we identified the most significant per gene for each test to produce the lists of Fluctuating and Directional SNP lists for downstream analyses (programs: python SNP.to.gene.py, Single.test.per.gene.py). The drift simulations were applied to each SNP independently (no linkage) because the tests are applied this way. While test outcomes may be correlated for closely related SNPs, this should make the thresholds conservative (Benjamini and Yekutieli, 2001).

### METHODS D: CORRELATIONS BETWEEN INTERVALS FOR CHANGE IN MINOR AND PERENNIAL ALLELES

Given ${\bar{z}}_{j}$ from 11 time points for each Fluctuating SNP, we calculated 10 *dz* intervals polarized so that change refers to the minor base. These values were used for Figure 2 with correlations calculated using the scipy stats package (within the program Minor.allele.change.Fluctuating.py). The average change and standard error (blue bars in Figure 3) were also obtained from this program. For the Perennial Allele analyses, we extracted the raw fastq files from the SRA archive (accessions in (Gould et al., 2017)) and then mapped these reads and called variants with same tools/settings applied to the IM pools and individuals (**Methods A**). At each Fluctuating SNP, we calculated the allele frequency in each pool as simply the reference count divided by the total count (requiring at least 20 reads to perform the calculation). We scored one of the bases as Perennial if the z in the Perennial pool was 0.4 greater than in the Annual pool. The program Peren.allele.change.Fluctuating.py performs the same calculations as Minor.allele.change.Fluctuating.py, except with alleles polarized as annual/perennial instead of major/minor (orange bars in Figure 3). Both programs calculate the $C_{g}$ statistic and test for associations with allele frequency.

### METHODS E: REMAPPING OF INBRED LINE SEQUENCE DATA, CALCULATING ADDITIVE EFFECTS, ASSOCIATION MAPPING, AND LD CALCULATIONS

Because the Illumina sequencing data on inbred lines from Troth et al. (2018) ascertained polymorphisms using a previous genome assembly, we here remapped the sequence data to the current *M. guttatus* reference genome for the 165 lines with phenotypic data from the greenhouse experiment. We used the same read mapping and SNP calling algorithms for these data as for the field population samples (**Methods A**). We then extracted all SNPs that were identified in the time series within the line vcf file. Requiring that each SNP is biallelic and that the same nucleotides segregate, we were able to score 1,765,940 of the time series SNPs in the lines (95%). These operations were executed using the programs bam.to.fastq.py, call.snps.by.section.py, relevant.snps.py, simplify.vcf.py, and order.snps.py.

Given the full genotype matrix, we calculated LD between SNPs at different levels of physical distance. We first calculated pairwise LD, and from this and allele frequencies, the r^2^ between all SNPs pairs within genes. We excluded any SNP pair where both loci were not called in at least 50 of the 165 lines. The average r^2^ at different levels of physical distance (bp between the two SNPs) were compiled for Supplemental Table 2. Next, we calculated LD by comparing each SNP in each a gene to all other SNPs in a distinct gene. This operation was first applied to neighboring genes and then to gene pairs separated by greater distance (from 50kb to 1mb within chromosomes). Finally, we randomly paired each gene to one on another chromosome and performed all pairwise comparison of SNPs (bottom row of Supplemental Table 2). Finally, we calculated the standardize LD for each gene (used for Figure 5 and associated tests) by first determining the average r^2^ between SNPs within genes at different inter‐SNP distances. We calculated means for six distance categories: <100bp, 100–199, 200–499, 500–999, 1000–1999,2000‐4999, and > = 5000bp. Given the whole genome means for each category, we determined the residual for each category within each gene (negative values indicating lower r^2^ for a given distance, positive values indicating higher r^2^). The standardized LD for each gene is a weighted average these residuals, which the weights equal to the number of inter‐SNP contrasts in that distance category. Dividing genes into quartiles provides the categories for Figure 4. These calculations were performed using LD_within_gene.py, LD_between_genes.py, and standardized_LD_per_gene.py.

Troth et al. (2018) determine the mean value for 13 traits using large greenhouse experiments on the 165 sequenced lines. There were two phenology measures, days to germination and days to flower. At the day of flowering, the 11 other traits were scored. Most traits are flower size dimensions (Anther Length, Corolla Length, Corolla Width, Stigma Length, Throat Width, Tube Length, flwrPC1, flwrPC2 ‐‐ the last two are principal components based on the first six). flwrPC1 is essentially a measure of overall flower size while flwrPC2 is determined by the width of corolla tube relative overall flower size. Fishman et al. (2002) provide a key for these dimensions. Troth et al. (2018) also scored the node producing the first flower, height from the ground to this first node, and the widest leaf. The widest leaf is a very strong positive predictor of total above‐ground biomass on the date that an IM plant flowers (Kelly, 2008).

Prior to association mapping, we used Beagle v5.4 (Browning et al., 2018) to impute missing genotypes and then reformatted the output using Make.gemma.gfile.py to make a bimbam input to Gemma (Zhou and Stephens, 2012). Next, we used Gemma to make a centered relatedness matrix from the full genotype matrix for the 165 lines, which was subsequently applied with a linear mixed model fit for each SNP to each trait. The [trait].assoc.txt output from Gemma provides the estimate and standard error for the average effect of each SNP on each trait. We then extracted the Fluctuating SNPs and determined the mean polygenic score for each year for each trait. Finally, we calculated the changes in these mean scores and compared them interval by interval to the changes in mean minor allele frequency and mean perennial allele frequency (Figure 2). After recording the covariance from real data (Table S3), we permuted the gene‐specific effect estimates across SNPs. This enforces the null hypothesis (no relationship between genotype to phenotype effect with change through time) and we recalculated the covariance after 10,000 permutations. The p‐value is the fraction of permuted covariances that exceed the observed value in absolute value (programs: gemma.effect.versus.greenness.py and polygenic.scores.fluctuators.py).

### METHODS F. TEMPORAL COVARIANCE CALCULATIONS, BOOTSTRAPPING, AND THE DISTRIBUTION OF CHANGE

The inputs to calculate the distribution of change and T_1_ and T_2_ are the transformed frequency and standard error ($z_{t}\;\mathrm{and}\;s_{t}$) at each SNP at each time point (Figure M1). $Var[\Delta z_{i}]$ in T_1_ is calculated as the raw variance in ($z_{y}-z_{x}$) minus the estimation error variance ($s_{y}^{2}+s_{x}^{2}$), where x and y are the time points for interval i and the calculations are performed over all SNPs. This calculation is applied to each time interval and the results summed to obtain T_1_. $Cov[\Delta z_{i},\Delta z_{j}]$ in T_2_ is the raw covariance plus an adjustment for estimation if the two intervals share a time point. Considering adjacent intervals where $\Delta z_{i}=$ $z_{y}-z_{x}$ and $\Delta z_{j}=$ $z_{x}-z_{u}$, the expected value for the raw covariance is the covariance of true values minus $s_{x}^{2}$. Thus, we add the average estimated $s_{x}^{2}$ to the raw covariances for all adjacent intervals. T_2_ is the sum across the values for all interval contrasts. As noted by Buffalo and Coop (2020), bootstrapping of genomic windows can be employed to place confidence bands on T_1_ and T_2_. We resampled 50kb windows of the genome in each bootstrap replicate of the current dataset, a value large enough to insure minimal LD between SNPs in distinct windows (Supplemental Table 2). For these calculations, we randomly assigned the “scored allele” – it was the reference base for half the SNPs and alternative base for the other half.

To create Figure 4 and associated results, we calculated the components of T_1_ and T_2_ for each gene. These values were then correlated to gene‐specific values of standardized LD (**Methods E**). The testing of whether correlations were non‐zero was also based on bootstrapping of 50kb windows (not treating each gene as independent). We also partitioned each bootstrap replicate to divide genes into the four LD categories of Figure 4 and recorded the category‐specific mean values of T_1_ and T_2_. The standard deviations of these values across 1000 bootstrap replicates provide the bands for Figure 4 . We wrote the programs temporal.covariance.all.py, temporal.covariance.bs.py, temporal.covariance.by.gene.py, and temporal.covariance.by.gene.bs.py to perform these calculations.

We applied ASHR to delta_z values from all single generation intervals from 2010–2017.  The input for each SNP/interval is the estimated delta_z and the p‐value for the test on whether this value is non‐zero.  With only two samples, the Directional and Fluctuating tests collapse to the same simple likelihood ratio test and the p‐value is obtained from the chi‐square distribution with 1 df.  The collection of estimates obtained here was also used to establish that the absolute magnitude of change is almost completely independent of allele frequency (Figure S3).  The calculations were performed using Delta_z.unpolarized.py and change.versus.maf.py. We implemented the R commands in ASHR using ashr1.r (this r script is contained in Supporting Information S1) that outputs the mixture distribution for change.  Our results depend only on the fraction of SNPs in each distribution (π) and the standard of the effect for each of these distributions.

## Supporting information

Supplemental Figure 1. The number of significant genes is strongly predicted by the total number of genes per chromosome.
Supplemental Figure 2. The distribution of Cg is reported for all Fluctuating SNPs.
Supplemental Figure 3. The arcsin squareroot transform effective normalizes allele frequency change.
Supplemental Figure 4. $Cov[\Delta z_{i},\Delta z_{j}]$ is calculated for each pair of distinct intervals (i and j) for all SNPs (yaxis) and the Fluctuating SNPs (x‐axis).

Appendix 1. The census population size of reproductive plants at Iron Mountain
Appendix 2: The change at neutral SNPs in each interval

Supplemental Table 1: The 1796 SNPs significant for fluctuating selection (A) and 40 SNPs significant for directional selection (B) are reported with parameter estimates from testing.
Supplemental Table 2. Linkage disequilibrium measured as r2 was calculated for SNPs in the time series analysis.
Supplemental Table 3. The covariances between mean allelic change statistics at Fluctuating SNPs (either minor alleles or perennial alleles) and mean polygenic scores are reported for each trait.
Supplemental Table 4. The covariance of changes, corrected for estimation error, for all pairwise comparisons of intervals.
Supplemental Table 5. The true change in dz (estimation error factored out) is estimated as a mixture of normal distributions by ASHR, all SNPs included.
Supplemental Table 6. The mean number of tests with a p‐value less than the cut‐off (reported in first column) are given for the neutral simulation in the second and third column.

Supplemental Information

Supplemental Information

## Data Availability

All sequence data has been submitted to the NCBI trace archive (SUB 12355869).
