# Supplementary material for: The genomic scale of fluctuating selection in a natural plant population
Source: Evol Lett. 2022 Dec 11;6(6):506–21. doi: 10.1002/evl3.308 (PMC9783439; doi:10.1002/evl3.308)
Supplement: Supplementary file 1 — Supplemental Figure 1. The number of significant genes is strongly predicted by the total number of genes per chromosome. Supplemental Figure 2. The distribution of Cg is reported for all Fluctuating SNPs. Supplemental Figure 3. The arcsin squareroot transform effective normalizes allele frequency change. Supplemental Figure 4. Cov[Δzi,Δzj] is calculated for each pair of distinct intervals (i and j) for all SNPs (yaxis) and the Fluctuating SNPs (x‐axis). [file EVL3-6-506-s001.pdf]

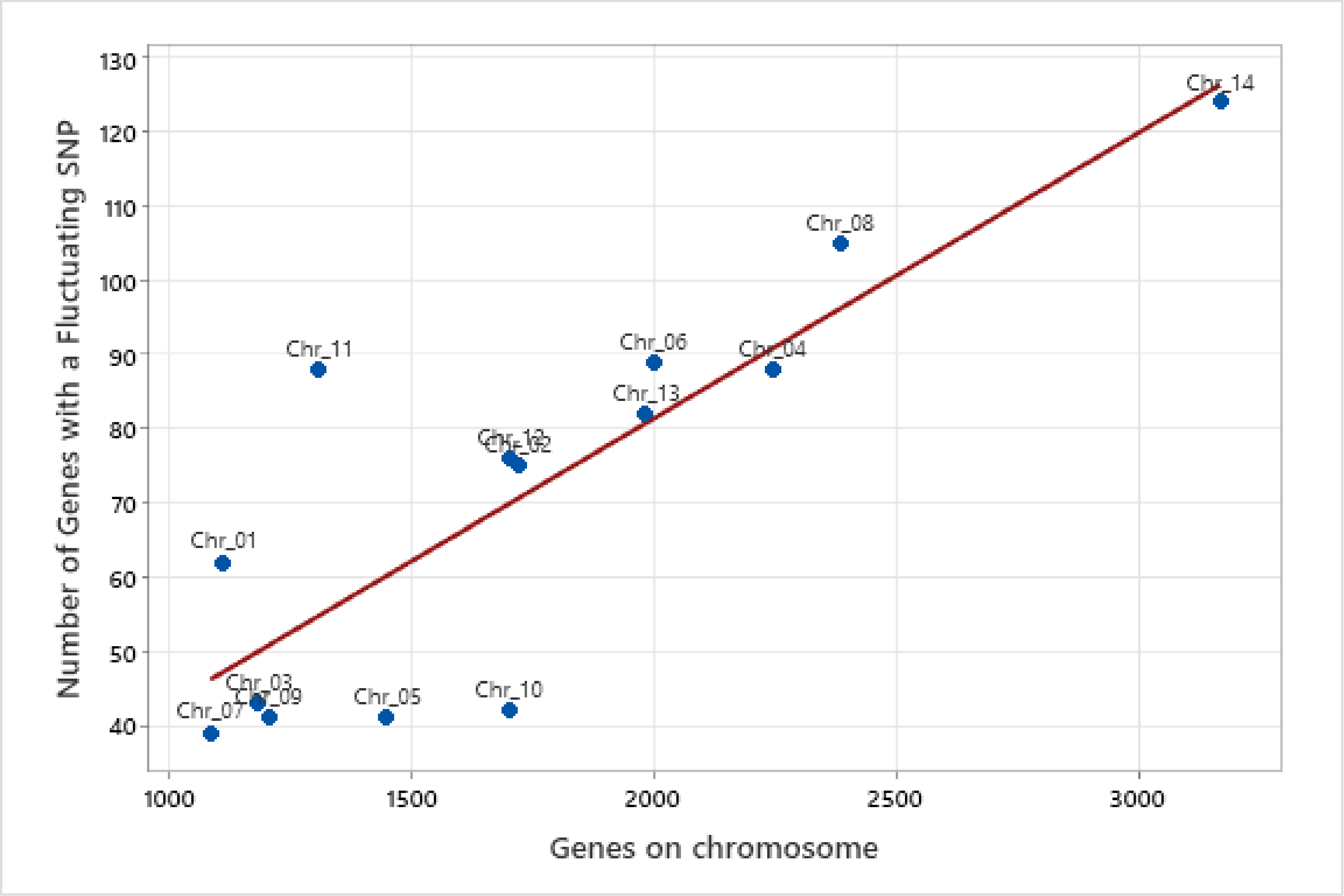

Supplemental Figure 1. The number of significant genes is strongly predicted by the total number of genes per chromosome. The large positive residual for Chr_11 is not due to meiotic drive locus. 65 of the 89 Fluctuating SNPs are outside the Drive region of this chromosome.

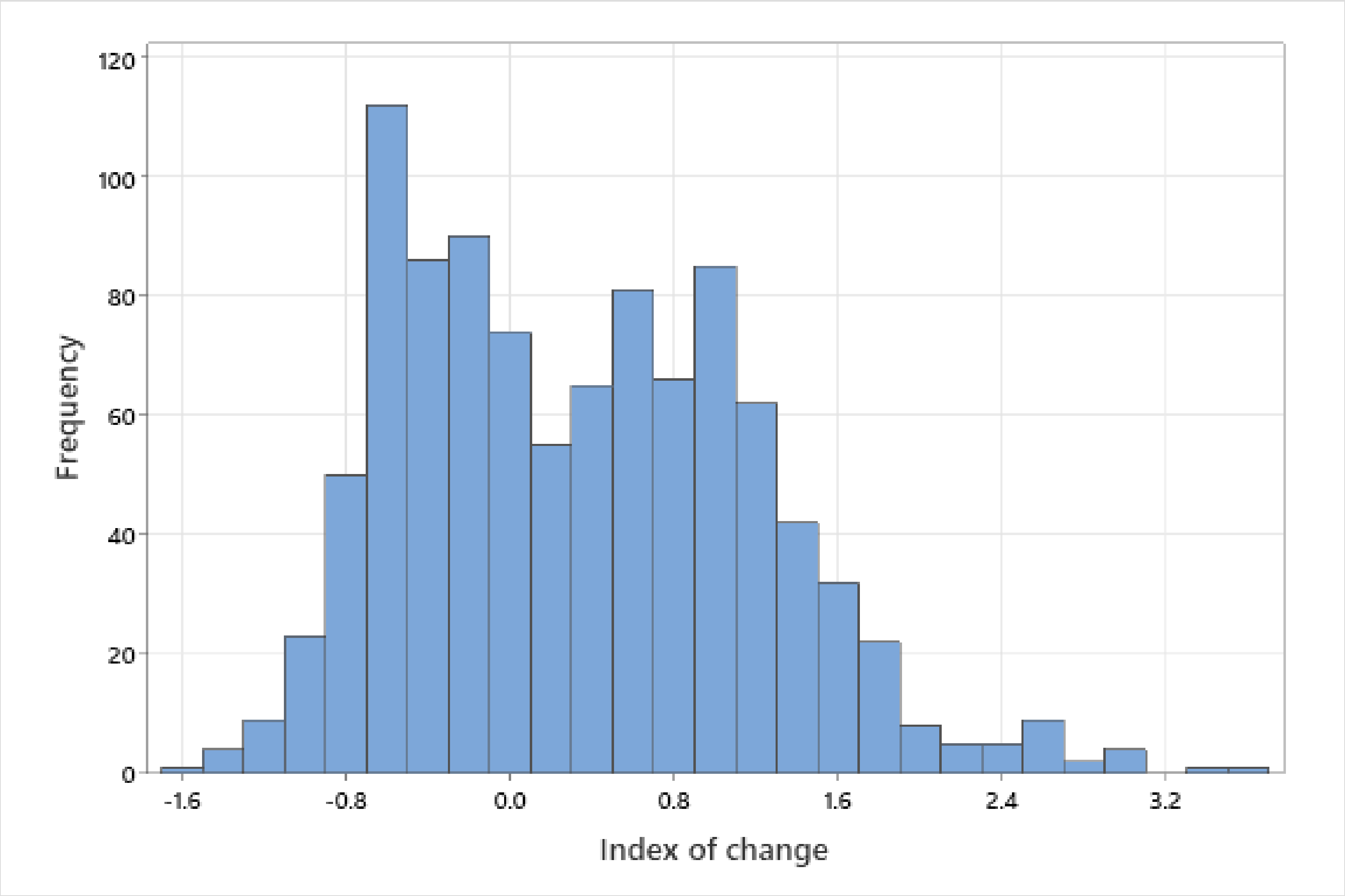

Supplemental Figure 2. The distribution of C_g is reported for all Fluctuating SNPs.

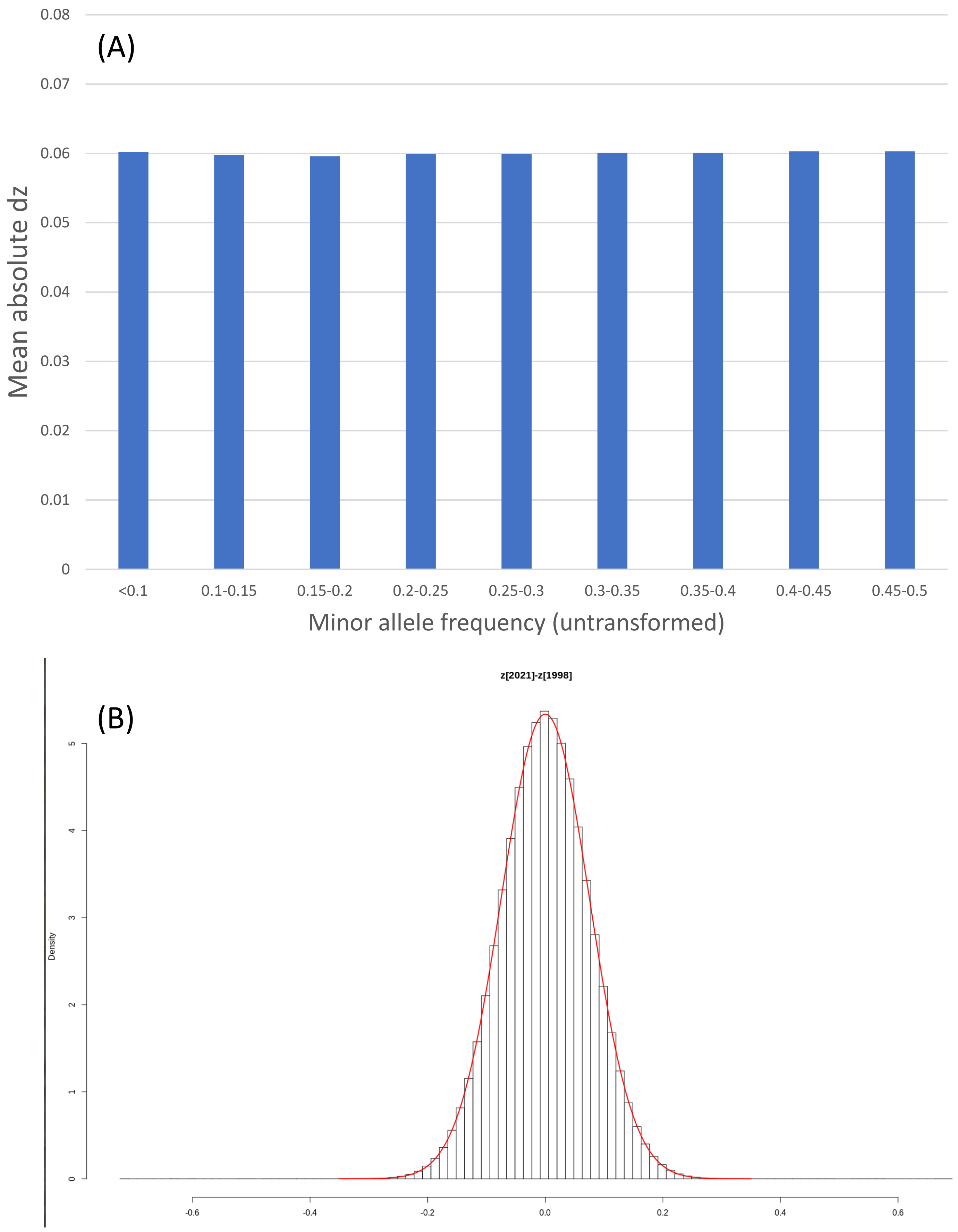

Supplemental Figure 3. The arcsin squareroot transform effective normalizes allele frequency change. (A) The absolute change in transformed allele frequency was determined for each SNP over each one-year interval from 2010 to 2017. The mean of change for z is plotted against untransformed minor allele frequency. (B) The distribution of changes in transformed allele frequency from 1998 to 2021 (all SNPs) is compared to the normal density function (red curve).

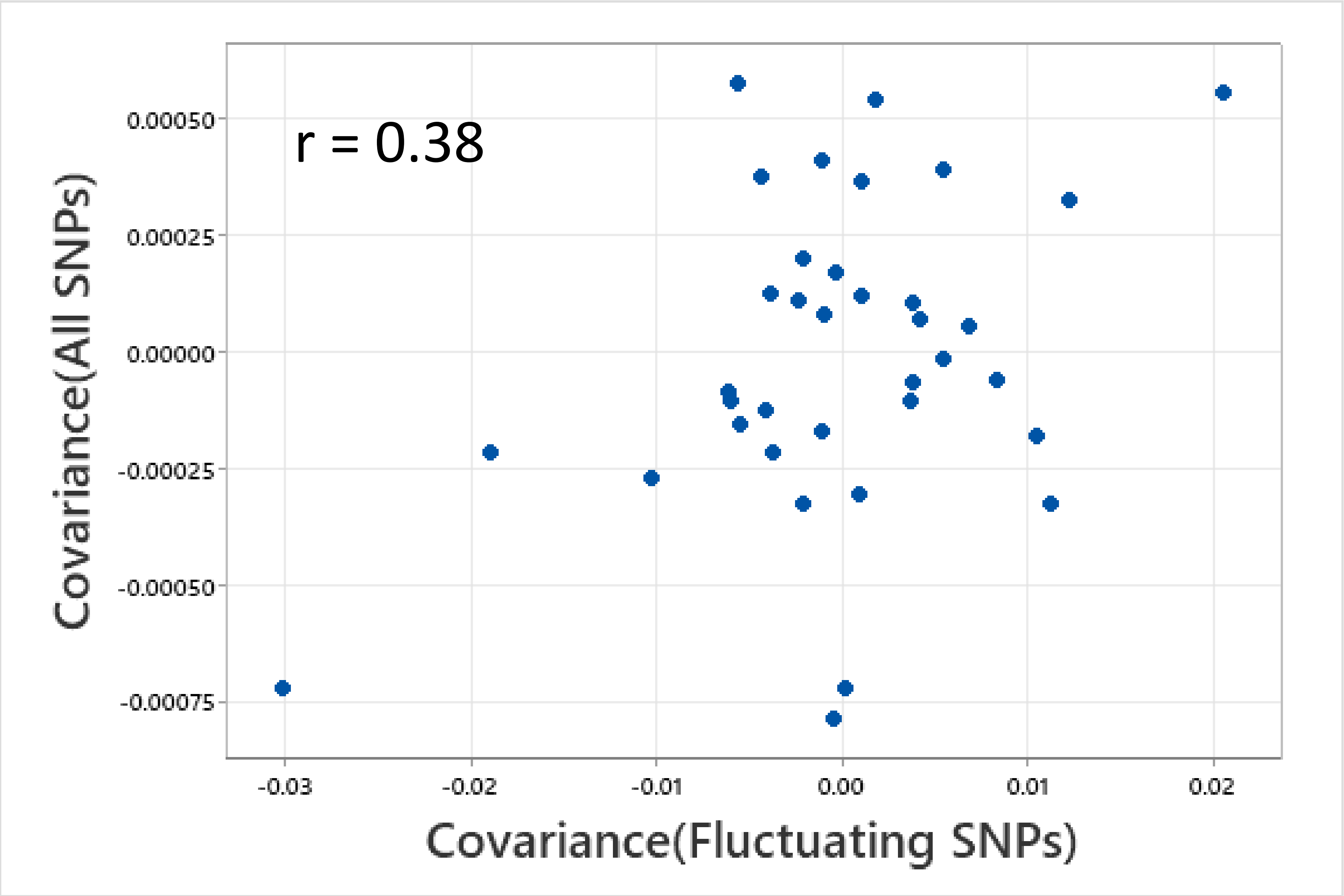

Supplemental Figure 4. $Cov[\Delta z_i, \Delta z_j]$ **is calculated for each pair of distinct intervals (i and j) for all SNPs (y-axis) and the Fluctuating SNPs (x-axis). The Pearson correlation is 0.38. The differing scales reflect the much larger magnitude of values for the Fluctuating SNPs (x-axis).**

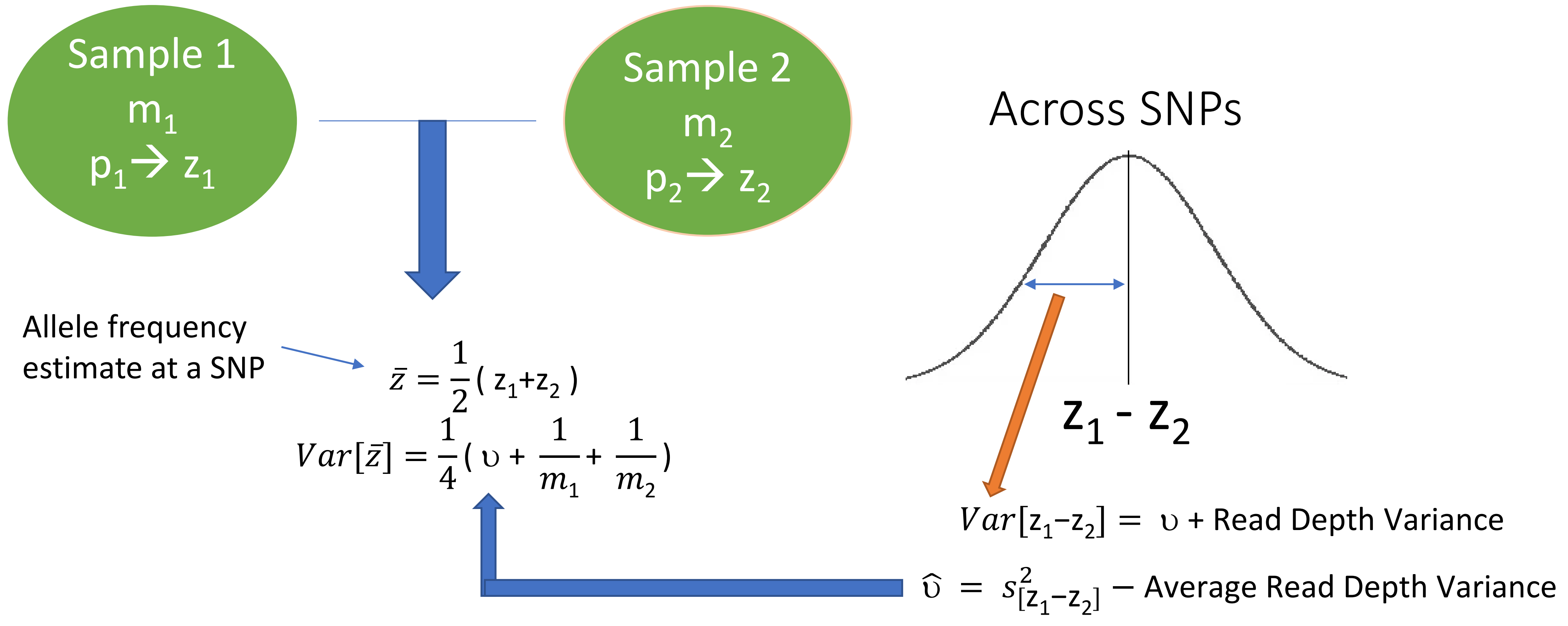

Figure M1. The estimate ($\bar{z}$) and the standard error ($\sqrt{Var[\bar{z}]}$) on allele frequency for a population is obtained from two replicate samples from each year. Here, p = untransformed reference base frequency, z = transformed frequency, m = read depth, and the subscripts 1 and 2 distinguish the two independent samples. The allele frequency estimate, $\bar{z}$, is the average of the pool specific estimates. The standard error on this estimate depends on the read depths (m) which are specific to this SNP as well as υ, which is the same for all SNPs in the genome. υ is determined by the number of genomes contributing to each pool and how evenly they contribute. If each sample is created by N diploid individuals that contribute equally to the DNA pool, then $\upsilon = \frac{1}{2N} + \frac{1}{2N} = \frac{1}{N}$. In this experiment N is very large, but we cannot be certain that all seeds contributed equally to each pool. For this reason, we estimate υ from the overall dispersion of $z_1 - z_2$ across the entire genome.
