# Supplementary material for: The genomic scale of fluctuating selection in a natural plant population
Source: Evol Lett. 2022 Dec 11;6(6):506–21. doi: 10.1002/evl3.308 (PMC9783439; doi:10.1002/evl3.308)
Supplement: Supplementary file 2 — Appendix 1. The census population size of reproductive plants at Iron Mountain Appendix 2: The change at neutral SNPs in each interval [file EVL3-6-506-s004.pdf]

Supplemental Information

Appendix 1. The census population size of reproductive plants at Iron Mountain

As part of a selection component experiment (1), Patrick Monnahan established 16 sampling quadrats across the field site at Iron Mountain. The area, approximately 180 m^2, contained nearly all *M. guttatus* plants that eventually reached flower at IM that year. The 10 cm^2 quadrats were uniformly located over the area. Patrick followed the plants within the quadrats until the end of the season and recorded the number of plants that successfully progressed to flower and the number that did not:

Quadrat	Plants that failed to flower	Plants that flowered
1	14	23
2	11	19
3	11	7
4	32	24
5	8	19
6	11	17
7	12	36
8	4	5
9	9	10
10	7	23
11	3	9
12	12	15
13	6	25
14	2	7
15	5	18
16	5	8
Mean	9.50	16.56

For flowering plants, the estimated number per square meter is 1656.25. The estimated total population size (flowering plants) is thus 298,125 with a 95% confidence interval (221,445 – 374,805). The number of flowering plants varies from year to year. This year (2013) was moderate in terms of density, but high for mean seedset of flowering plants (2).

Appendix 2: The change at neutral SNPs in each interval

To determine if genomewide allele frequency change was greatly elevated in some intervals relative to others, we modified the program that estimates N_e from the full timeseries (Ne.robust.estimation.py, **Methods B**) to treat change between successive population samples. For each interval, e.g. 2010 to 2011, we calculated the interquartile range for SNP specific differences in z, as well as the estimation error variance. The IQR is used to estimate the total variance in change, the variance in true divergence

is subsequently obtained by subtracting the estimation error from the total variance (**Methods B**). The expected value for the true variance is t Var[Δz], where t is the number of generations in the interval and Var[Δz] is the variance in change per generation. Since E[Δz]=0.0, we use Sqrt(Var[Δz]/t) as a measure of the magnitude of change per generation. We applied this calculation separately to each chromosome for each interval. The average change and the S.E.M. are report below. The program used to perform these calculations, Change.per.interval.py, is in Supplemental File 1.

start year	end year	t	Mean change per generation (across chromosomes)	Standard Error (across chromosomes)
1998	2007	9	0.0081	0.0005
2007	2010	3	0.0137	0.0009
2010	2011	1	0.0170	0.0017
2011	2012	1	0.0070	0.0019
2012	2013	1	0.0082	0.0016
2013	2014	1	0.0277	0.0016
2014	2015	1	0.0250	0.0027
2015	2016	1	0.0238	0.0019
2016	2017	1	0.0000	0.0000
2017	2021	4	0.0173	0.0002

The first noteworthy result is the 0.0 value for change in 2016-2017. For this interval, the total divergence in z was actually slightly less than predicted by estimation error. Second, the two intervals that might be expected to show greatly elevated change do not. Severe drought events limited reproduction of plants in 2015 and 2019 (3, 4). As a consequence, bottlenecks might have produced larger magnitude changes from 2015 to 2016 and from 2017 to 2021. Yet neither interval is extreme. This is consistent with the observation that the flowering plants in the second year of each interval (2016 and 2020) were not clearly lower than in other years (Nic Kooyers, personnel communication). Perhaps more importantly, there are no high change outliers across intervals. Interestingly, there is a tendency for the average change per year to be slightly lower in the multi-generation intervals (1998-2007, 2007-2010, and 2017-2021). This may be a simple consequence of the negative covariance of changes between generations for neutral SNPs (e.g. Figure 4).

Cited in Appendices

1. P. J. Monnahan, J. Colicchio, L. Fishman, S. J. Macdonald, J. K. Kelly, Predicting evolutionary change at the DNA level in a natural Mimulus population. *PLOS Genetics* **17**, e1008945 (2021).
2. Y. W. Lee, L. Fishman, J. K. Kelly, J. H. Willis, A Segregating Inversion Generates Fitness Variation in Yellow Monkeyflower (Mimulus guttatus). *Genetics* **202**, 1473-1484 (2016).
3. A. Troth, J. R. Puzey, R. S. Kim, J. H. Willis, J. K. Kelly, Selective trade-offs maintain alleles underpinning complex trait variation in plants. *Science* **361**, 475-478 (2018).
4. N. J. Kooyers *et al.*, Population responses to a historic drought across the range of the common monkeyflower (Mimulus guttatus). *American Journal of Botany* **108**, 284-296 (2021).
