# Supplementary material for: The genomic scale of fluctuating selection in a natural plant population
Source: Evol Lett. 2022 Dec 11;6(6):506–21. doi: 10.1002/evl3.308 (PMC9783439; doi:10.1002/evl3.308)
Supplement: Supplementary file 4 — Supplemental Information [file EVL3-6-506-s005.pdf]

Supplemental Tables

Supplemental Table 1: The 1796 SNPs significant for fluctuating selection (A) and 40 SNPs significant for directional selection (B) are reported with parameter estimates from testing. Both lists were thinned to one test per gene for subsequent testing.
Excel file

Supplemental Table 2. Linkage disequilibrium measured as r^2 was calculated for SNPs in the time series analysis. "Type" is the nature of contrast between SNPs. For the Inter-SNP distances of 50kb and greater, we contrasted each gene to the next one downstream on a chromosome at that specified distance.

Inter-SNP distance	Type	Number of SNP pairs	Mean r^2
<100	Within genes	8190844	0.388
100-200	Within genes	5989738	0.355
200-500	Within genes	15692989	0.332
500-1000	Within genes	20804591	0.313
1000-2000	Within genes	27883616	0.300
2000-5000	Within genes	30318743	0.301
1000-2000	Neighboring genes	3178100	0.203
2000-5000	Neighboring genes	21438769	0.157
5000-10000	Neighboring genes	23573864	0.128
10000-20000	Neighboring genes	12519962	0.106
50000		114648600	0.027
100000		112122337	0.022
200000		108922776	0.018
500000		103924828	0.016
1000000		98740434	0.016
Different chromosomes		55815358	0.013

Supplemental Table 3. The covariances between mean allelic change statistics at Fluctuating SNPs (either minor alleles or perennial alleles) and mean polygenic scores are reported for each trait. The covariance is taken over all 10 intervals of change. The p-values are the fraction of covariances from 10000 permutations of genotypic effects that exceed the observed values in absolute value.

Trait	Minor allele change versus Change in polygenic score		Perennial allele change versus Change in polygenic score	
	Covariance	p-value	Covariance	p-value
Anther Length	3.3656E-05	0.1701	3.8842E-05	0.1452
Corolla Length	6.7403E-05	0.1624	6.3692E-05	0.2317
Corolla Width	0.00012257	0.0255	0.00013406	0.0247
Days to Flower	5.7019E-05	0.1195	5.6023E-05	0.1653
flwrPC1	4.0125E-05	0.0574	4.223E-05	0.0678
flwrPC2	2.2819E-05	0.0934	2.696E-05	0.0732
Germination date	4.4984E-05	0.0552	4.6802E-05	0.0668
Height to first node	-0.0009017	0.0431	-0.0011326	0.0214
Node	5.2658E-06	0.5053	5.1758E-06	0.545
Stigma Length	4.2163E-05	0.1059	5.165E-05	0.0703
Throat Width	3.7723E-05	0.036	4.3734E-05	0.0271
Tube Length	2.7018E-05	0.3388	2.8868E-05	0.3501
Widest Leaf	0.00026541	0.0023	3.55E-04	0.0004

Supplemental Table 4. The covariance of changes, corrected for estimation error, for all pairwise comparisons of intervals. The 95% Confidence Bounds were obtained by bootstrapping.

Interval1	Inverval2	Covariance	Lower Bound	Upper Bound	Significance
1998	2007	-0.000357	-0.000419	-0.000296	sig_negative
1998	2010	0.000364	0.000315	0.000413	sig_positive
1998	2011	-0.000156	-0.000195	-0.000117	sig_negative
1998	2012	-0.000016	-0.000053	0.000022	NS
1998	2013	-0.000328	-0.000389	-0.000266	sig_negative
1998	2014	0.000322	0.000251	0.000394	sig_positive
1998	2015	0.000575	0.000513	0.000638	sig_positive
1998	2016	-0.000327	-0.000373	-0.000277	sig_negative
1998	2017	-0.000723	-0.000777	-0.000668	sig_negative
2007	2010	-0.000572	-0.000631	-0.000514	sig_negative
2007	2011	0.000106	0.000072	0.000140	sig_positive
2007	2012	-0.000128	-0.000164	-0.000093	sig_negative
2007	2013	0.000537	0.000481	0.000598	sig_positive
2007	2014	-0.000274	-0.000338	-0.000209	sig_negative
2007	2015	-0.000789	-0.000850	-0.000730	sig_negative
2007	2016	0.000373	0.000327	0.000424	sig_positive
2007	2017	0.000556	0.000502	0.000607	sig_positive
2010	2011	-0.000071	-0.000107	-0.000039	sig_negative
2010	2012	0.000078	0.000046	0.000112	sig_positive
2010	2013	-0.000306	-0.000358	-0.000258	sig_negative
2010	2014	0.000109	0.000056	0.000165	sig_positive
2010	2015	0.000388	0.000338	0.000438	sig_positive
2010	2016	-0.000217	-0.000257	-0.000177	sig_negative
2010	2017	-0.000174	-0.000219	-0.000130	sig_negative
2011	2012	-0.000096	-0.000121	-0.000069	sig_negative
2011	2013	0.000120	0.000082	0.000160	sig_positive
2011	2014	-0.000087	-0.000135	-0.000039	sig_negative
2011	2015	-0.000106	-0.000143	-0.000066	sig_negative
2011	2016	0.000121	0.000091	0.000151	sig_positive
2011	2017	0.000055	0.000021	0.000090	sig_positive
2012	2013	-0.000207	-0.000254	-0.000163	sig_negative
2012	2014	0.000067	0.000025	0.000110	sig_positive
2012	2015	0.000200	0.000160	0.000238	sig_positive
2012	2016	-0.000064	-0.000095	-0.000031	sig_negative
2012	2017	-0.000109	-0.000146	-0.000073	sig_negative
2013	2014	-0.000385	-0.000478	-0.000294	sig_negative
2013	2015	-0.000724	-0.000784	-0.000659	sig_negative
2013	2016	0.000407	0.000360	0.000452	sig_positive

2013	2017	0.000170	0.000114	0.000225	sig_positive
2014	2015	-0.000133	-0.000215	-0.000053	sig_negative
2014	2016	-0.000059	-0.000111	-0.000005	sig_negative
2014	2017	-0.000219	-0.000282	-0.000155	sig_negative
2015	2016	-0.000043	-0.000095	0.000010	NS
2015	2017	-0.000184	-0.000242	-0.000133	sig_negative
2016	2017	0.000075	0.000030	0.000120	sig_positive

Supplemental Table 5. The true change in dz (estimation error factored out) is estimated as a mixture of normal distributions by ASHR, all SNPs included. The mean of each normal distribution is zero. The standard deviation differs between the six sub-distributions.

proportion of SNPs	Standard Deviation of change (dz)
0.328	0
0.441	0.004
0.173	0.0319
0.059	0.0451
7.48E-05	0.1804
3.61E-06	0.2551

Supplemental Table 6. The mean number of tests with a p-value less than the cut-off (reported in first column) are given for the neutral simulation in the second and third column. These are very similar among tests, which is expected because each yields a very uniform distribution of p-values. The actual number of tests passing each threshold in the data is reported in the fourth and fifth columns. Since the "significant" tests in columns 2 and 3 are false discoveries (the null hypothesis is true), the values of column 4 relative to column 2 (and column 5 relative to column 3) provide an empirical false discovery rate as a function of p-value. The highlighted rows (green for Directional, orange for Fluctuating) correspond to an FDR<0.05.

	Neutral simulations (Ne = 11790)		Real data		False Discovery Rate	
Test>>	Directional	Fluctuating	Directional	Fluctuating	Directional	Fluctuating
Pvalue_cutoff						
1.0E-10	0	0	3	152	0	0
2.0E-10	0	0	3	166	0	0
3.0E-10	0	0	3	169	0	0
4.0E-10	0	0	3	174	0	0
5.0E-10	0	0	4	180	0	0
6.0E-10	0	0	4	186	0	0
7.0E-10	0	0	4	195	0	0
8.0E-10	0	0	4	197	0	0
9.0E-10	0	0	5	201	0	0
1.0E-09	0	0	5	206	0	0
2.0E-09	0	0	6	226	0	0
3.0E-09	0.01	0	8	236	1.25E-03	0.00E+00
4.0E-09	0.01	0.01	8	251	1.25E-03	3.98E-05
5.0E-09	0.01	0.01	8	258	1.25E-03	3.88E-05
6.0E-09	0.01	0.01	10	263	9.99E-04	3.80E-05
7.0E-09	0.02	0.01	11	267	1.81E-03	3.75E-05
8.0E-09	0.02	0.01	11	269	1.81E-03	3.72E-05
9.0E-09	0.03	0.01	12	274	2.49E-03	3.65E-05
1.0E-08	0.03	0.02	12	277	2.49E-03	7.22E-05
2.0E-08	0.05	0.04	14	311	3.56E-03	1.29E-04
3.0E-08	0.07	0.05	17	327	4.10E-03	1.53E-04
4.0E-08	0.09	0.06	18	346	4.98E-03	1.73E-04
5.0E-08	0.12	0.09	20	358	5.96E-03	2.51E-04
6.0E-08	0.14	0.1	20	369	6.95E-03	2.71E-04
7.0E-08	0.16	0.11	20	377	7.94E-03	2.92E-04
8.0E-08	0.18	0.14	22	387	8.12E-03	3.62E-04
9.0E-08	0.2	0.16	23	391	8.62E-03	4.09E-04
1.0E-07	0.22	0.17	23	399	9.47E-03	4.26E-04
2.0E-07	0.42	0.37	26	466	1.59E-02	7.93E-04
3.0E-07	0.6	0.56	28	502	2.10E-02	1.11E-03
4.0E-07	0.79	0.74	30	526	2.57E-02	1.40E-03
5.0E-07	1	0.94	33	544	2.94E-02	1.72E-03
6.0E-07	1.2	1.12	35	563	3.31E-02	1.99E-03

7.0E-07	1.39	1.31	37	578	3.62E-02	2.26E-03
8.0E-07	1.56	1.49	37	591	4.05E-02	2.51E-03
9.0E-07	1.74	1.65	37	610	4.49E-02	2.70E-03
1.0E-06	1.93	1.83	40	626	4.60E-02	2.91E-03
2.0E-06	3.77	3.67	50	719	7.01E-02	5.08E-03
3.0E-06	5.62	5.65	57	773	8.97E-02	7.26E-03
4.0E-06	7.5	7.5	62	828	1.08E-01	8.98E-03
5.0E-06	9.34	9.35	65	875	1.26E-01	1.06E-02
6.0E-06	11.23	11.15	68	905	1.42E-01	1.22E-02
7.0E-06	13.18	13.03	74	933	1.51E-01	1.38E-02
8.0E-06	14.99	14.94	81	970	1.56E-01	1.52E-02
9.0E-06	16.87	16.81	82	1000	1.71E-01	1.65E-02
1.0E-05	18.67	18.73	84	1047	1.82E-01	1.76E-02
2.0E-05	37.22	37.12	117	1291	2.41E-01	2.79E-02
3.0E-05	55.7	55.63	140	1492	2.85E-01	3.59E-02
4.0E-05	74.09	74.12	166	1655	3.09E-01	4.29E-02
5.0E-05	92.54	92.71	196	1796	3.21E-01	4.91E-02
6.0E-05	111	111.27	217	1943	3.38E-01	5.42E-02
7.0E-05	129.44	129.71	234	2068	3.56E-01	5.90E-02
8.0E-05	147.91	148.28	254	2182	3.68E-01	6.36E-02
9.0E-05	166.48	166.78	281	2300	3.72E-01	6.76E-02
